# In vitro and in vivo analyses of co-infections with peste des petits ruminants and capripox vaccine strains

**DOI:** 10.1186/s12985-021-01539-7

**Published:** 2021-04-07

**Authors:** Dajun Zhang, Bo Yang, Ting Zhang, Xijuan Shi, Chaochao Shen, Haixue Zheng, Xiangtao Liu, Keshan Zhang

**Affiliations:** State Key Laboratory of Veterinary Etiological Biology, National Foot-and-Mouth Disease Reference Laboratory, Lanzhou Veterinary Research Institute, Chinese Academy of Agriculture Science, Lanzhou, 73004 People’s Republic of China

**Keywords:** In vitro and in vivo, Peste des petits ruminants, Capripox, Vaccine strains, Coinfection, Evaluation

## Abstract

**Background:**

Peste des petits ruminants (PPR) and goat pox (GTP) are two devastating animal epidemic diseases that affect small ruminants. Vaccination is one of the most important measures to prevent and control these two severe infectious diseases.

**Methods:**

In this study, we vaccinated sheep with PPR and POX vaccines to compare the changes in the antibody levels between animals vaccinated with PPRV and POX vaccines alone and those co-infected with both vaccines simultaneously. The cell infection model was used to explore the interference mechanism between the vaccines in vitro. The antibody levels were detected with the commercial ELISA kit. The Real-time Quantitative PCR fluorescent quantitative PCR method was employed to detect the viral load changes and cytokines expression after the infection.

**Results:**

The concurrent immunization of GTP and PPR vaccine enhanced the PPR vaccine's immune effect but inhibited the immune effect of the GTP vaccine. After the infection, GTP and PPR vaccine strains caused cytopathic effect; co-infection with GTP and PPR vaccine strains inhibited the replication of PPR vaccine strains; co-infection with GTP and PPR vaccine strains enhanced the replication of GTP vaccine strains. Moreover, virus mixed infection enhanced the mRNA expressions of TNF-α, IL-1β, IL-6, IL-10, IFN-α, and IFN-β by 2–170 times. GTP vaccine strains infection alone can enhanced the mRNA expression of IL-1β, TNF-α, IL-6, IL-10, while the expression of IFN-α mRNA is inhibited. PPR vaccine strains alone can enhanced the mRNA expression of IFN-α, IFN-β, TNF-α, and has little effect the mRNA expression of IL-1β, IL-6 and IL-10. The results showed that GTP and PPR vaccine used simultaneously in sheep enhanced the PPR vaccine's immune effect but inhibited the immune effect of the GTP vaccine in vivo. Furthermore, an infection of GTP and PPR vaccine strains caused significant cell lesions in vitro; co-infection with GTP + PPR vaccine strains inhibited the replication of PPR vaccine strains, while the co-infection of GTP followed by PPR infection enhanced the replication of GTP vaccine strains. Moreover, virus infection enhanced the expressions of TNF-α, IL-1β, IL-6, IL-10, IFN-α, and IFN-β.

**Conclusions:**

Peste des petits ruminants and capripox vaccine strains interfere with each other in vivo and vitro.

## Background

Peste des petits ruminants (PPR) is defined by the World Organisation for Animal Health (OIE) as a Class A fulminating infectious disease. It is a highly contagious acute viral disease that seriously affects sheep and goats and has a huge impact on the economy [1]. PPR was first described in Western Africa in 1942 [2], after which the peste des petits ruminants virus (PPRV) was isolated from sheep embryonic kidney cells [3]. The first PPR case in goats in China was reported in 2007 [4]. The incubation period of PPRV is 2–7 days, and the main clinical manifestations of PPR include fever, tears, and snot, stomatitis, pneumonia, and diarrhea [5]. The disease endemic in many parts of the world, especially in sheep farming areas of Africa, Middle East, Asia. [6]. Susceptible animals can be directly infected or by inhalation [7]. There are currently no reports on arthropods as its vector; thus, PPRV is believed to be transmitted through aerosols or contaminated gas [8].

Goat pox (GTP) is a viral infection disease that seriously endangers the growth of goats/sheep. It is an acute, febrile, and contagious disease caused by the Capripox virus [9, 10]. In goats/sheep, it is clinically characterized by elevated temperature, systemic papules or nodules, blisters, visceral lesions, and especially obvious pulmonary lesions [11, 12]. As the principal host, goats/sheep of all ages are affected by this disease. Yet, death primarily occurs in lambs, and adult goats/sheep [13]. This disease is frequent in Asia, Africa, the Middle East, and part of Europe, but it is also reported in many other parts of the world [14]. GTP is probably the most serious contagious disease in ruminants [15], which leads to substantial economic losses, reduces productivity and the quality of wool and leather products, and significantly impacts animal husbandry in epidemic areas [16].

The high infectiousness, high morbidity, and high mortality of PPR and GTP in small ruminants cause huge economic losses. A previous study found that the high mortality of sheep and goats' infected flock might be attributed to the co-infection's exacerbation effect by PPRV and GTPV [17]. The main means of preventing and controlling epidemic diseases are vaccine immunity [18, 19]. PPR and POX vaccines are two primary vaccines used for the immunization procedures. The breeding cost has increased due to many types of vaccines available on the market, the tedious immunization procedures, and the long time and labor required for vaccination. The administration of the two vaccines at the same time can save time and labor, reduce the breeding cost, and dramatically simplify the vaccine immunization procedures and increase animal welfare. It remains unknown what changes in antibody levels are induced by the combined use of the two vaccines, whether there is any interference between the two vaccines, and what is the underlying molecular interference mechanism. Previous studies have shown that PPRV and GTPV can be replicated in African green monkey kidney cells (Vero cells) [20, 21]. The study of virus-infected cells is essential for understanding the interference mechanism between viruses and their immunology. In addition, the interaction between the two viruses at the molecular level remains unclear.

In this study, sheep were vaccinated with PPR and POX vaccines to compare the changes in the antibody levels. Moreover, an in vitro cell infection model was constructed. TCID_50_ and RT-qPCR were used to detect the titer and viral nucleic acid load of each virus, as well as the changes in the mRNA transcription levels of factors related to the cellular immune effect so as to define the interference mechanism for in-vitro replication of PPR and POX vaccine strains, develop and improve scientific and rational immunization procedures, and provide a reference for guiding production practice.

## Materials and methods

### Vaccines, attenuated vaccine strains, and cells

Live PPR vaccines (Nigeria 75/1 Strain) and goat pox vaccines were obtained from China's domestic commercial market Goat skin fibroblast (GSF) cells and Vero cells were obtained from the Foot-and-Mouth Disease Epidemiology Team, Lanzhou Veterinary Research Institute, Chinese Academy of Agricultural Sciences. Both cells were cultured in the DMEM medium (Gibco) supplemented with 10% fetal calf serum (FBS; Gibco) and 1% penicillin and streptomycin (Harbin Pharmaceutical Group General Pharmaceutical Factory) in a humidified atmosphere containing 5%CO_2_/95% air at 37ºC. PRR vaccine strains and GTP vaccine strains were isolated from the vaccines mentioned above.

### Experimental design

#### In-vivo experiment

Ninety-five healthy fattening sheep (two months old) with negative PPR and POX antibodies, similar weight, normal growth, and breeding, were obtained from a sheep farm in northwest China. All sheep were kept under the same conditions. Animals were randomly divided into three groups: 32 sheep in Group A were vaccinated with the PPR vaccine, 32 sheep in Group B were vaccinated with the GTP vaccine, and 31 sheep in Group C were vaccinated with both vaccines simultaneously. Blood was separately collected on days 7, 14, 21, and 28 after the immunization and sent to the laboratory for antibody detection (Table 1). All animal studies were done in compliance with the regulations and guidelines of Lanzhou Veterinary Research Institute (Chinese Academy of Agriculture Science) institutional animal care and conducted according to the AAALAC and the IACUC guidelines (License No. SYXK [GAN] 2014–003).

**Table 1:** Grouping and treatment of experimental sheep

Group	Number	Immunization type and blood collection time			
		7d	14d	21d	28d
A	32	PPR vaccine			
B	32	Goat pox vaccine			
C	31	PPR vaccine + goat pox vaccine			

Day 0, day of immunization; blood was collected on day 7, 14, 21, and 28 after the immunization

#### In-vitro experiment

GSF cells were used in functional studies (viral replication assays), whereas vero cells were used for virus production and virus titration. GSF cells grown on a 6-well culture plate were divided into six groups, including the group infected with PPRV alone, the group infected with GTPV alone, the group co-infected with PPRV and GTPV, the group infected with PPRV followed by GTPV (GTPV was inoculated 3 h later), the group infected with GTPV followed by PPRV (PPRV was inoculated 3 h later), and the control group. Each group was inoculated with 0.5 MOI PPRV and GTPV according to the requirements, and the control group was inoculated with an equal volume of cell culture solution (Table 2). The cells were then placed in a 37 °C incubator and were harvested 36 h later, which was long enough for viruses to replicate. This time duration also allowed cells to be harvested before the viruses destroyed the entire GSF monolayer cells and before the viral titer began to drop.

**Table 2:** Cell monolayers were inoculated at a multiplicity of infection of 0.5 for each virus either singly (infection), or in combination

Infection type	Infection order		Infection MOI		Infection title
	First	Second	0 h	3 h	
Infection	PPRV		0.5	**–**	PPRV
Superinfection	PPRV	GIPV	0.5	0.5	PPRV 1st GTPV 2nd
Coinfection	PPRV + GTPV		1	**–**	Coinfection
Superinfection	GTPV	PPRV	0.5	0.5	GTPV 1st PPRV 2nd
Infection	GTPV		0.5	**–**	GTPV

Mixed viral infections resulted from inoculation with both viruses at the same time (coinfection) or from single inoculations occurring 3 h apart (superinfection)

“–“ means no virus infection

An inverted fluorescence microscope was used for observation and photographing. The cells were then stored at − 80 °C. Real-time detection was performed on the viral titer and mRNA levels of cellular immune factors in each group to compare differences between them. Each cell line-virus inoculation combination was repeated three times.

### Detection of antibody levels in animal serum

#### ELISA

PPR antibody detection was conducted according to the instructions on the PPR antibody detection kit of this laboratory. The results were determined as follows: Blocking rate (PI) = (1—Sample value/Mean of negative control wells) × 100%; PI ≥ 50% suggests positive and PI < 50% suggests negative. GTP antibody detection was conducted according to the instructions on the GTP antibody detection kit provided by the Diagnosis Center of Lanzhou Veterinary Research Institute, Chinese Academy of Agricultural Sciences. The results were judged according to the following criteria: OD450 ≥ 0.25 indicated positive antibody; OD450 ≤ 0.2 negative antibody; 0.25 > OD450 > 0.20 suspicious positive, after which, the detection was repeated; OD450 ≥ 0.23 was considered positive and OD450 ≤ 0.23 was considered negative.

#### VNT detection

All tested serum samples including positive and negative control serum were incubated at 56 °C for 30 min. To assess the titer of PPRV、GTPV specific antibody, triplicates of tested serial twofold dilution series were titrated against a constant titer of PPR、GTP vaccine strain 100TCID_50_. The tested serum, positive and negative control serum were diluted in DMEM without FBS (from 1:10 to 1:1280). Serum dilutions and the fixed amount of virus strain were incubated for 2 h at 37 °C. After the neutralization step 100 μL suspension of Vero cells in DMEM with 10% FBS was added to each well. In each test one control plate was included with only virus titration and titration of positive and negative control serum. Plates were incubated at 37 °C, 5% CO2. After 4 days plates were examined for appearance of cytopathogenic effect (CPE) and final reading was taken on day 7. Results were recorded and titer was calculated using the Spearman and Kaerber method. Samples with an antibody titer ≥ 1:10 were considered as positive.

### Viral nucleic acid extraction and quantitative real-time PCR detection

GTPV-infected GSF cell samples were collected, and virus DNA was extracted by following the instructions on the virus DNA extraction kit of OMEGA US. Transfer 200 μL of sample solution to a centrifuge tube for viral DNA extraction. Capripoxvirus-specific primers and probes recommended on the official website of OIE were used [22], including forward primer: 5′-AAA ACG GTA TAT GGA ATA GAG TTG GAA-3′, reverse primer:5′-AAA TGA AAC CAA TGG ATG GGA TA-3′, probe primer: 5′-FAM-TGG CTC ATA GAT TTC CT-MGBNFQ-3′. Reactions occurred in the optical 96-well reaction plate (Applied Biosystems), and the following amplification programs were applied: 50 °C 2 min; 95 °C 10 min; 45 cycles at 95 °C for 15 s and at 60 °C for 1 min.

PPRV-infected GSF cell samples were collected, and the total cell RNA was extracted with the TRIzol (Invitrogen) method. The primers and probes specific for PPRV detection recommended on the official website of OIE were used [23], including forward primer: 5′-GAG TCT AGT CAA AAC CCT CGT GAG-3′, reverse primer: 5′-TCT CCC TCC TCC TGG TCC TC-3′, probe primer: 5′-FAM-CGG CTG AGG CAC TCT TCA GGC TGC-BHQ1-3′. One-step RT-PCR kits were used for detection, the reaction system was: 2 × one-step RT-PCR Buffer III 12.5 μL, 0.5 μL TaKaPa EX Taq HS 0.5 μL, 0.5 μL Primer Script RT Enzyme mix II 0.5 μL, upstream primers 0.5 μL, downstream primers 0.5 μL, probe 1 μL, RNA template 2 μL, and DEPC water was added to 25 μL. Reaction program: 42 °C 10 min, 94 °C 10 s, 57 °C 30 s, and 72 °C 30 s, 40 cycles in total.

### Real-time PCR detection of mRNA of cellular immune-related effect factors

Total RNA of the cells infected with PPRV and GTPV was extracted with the TRIzol method, cDNA was obtained by reverse transcription using RNA as the template, and relative quantitative detection was performed by using the expression levels of GAPDH mRNA as internal parameter values. Reverse transcription system 10 μL: 2 μL 5 × Prime script RT Master Mix, 6 μL RNase Free ddH2O, and 2μL RNA template. Reaction program: 37 °C 15 min and 85 °C 5 s. Relative quantitative reaction system (20 μL): 10 μL SYBR Permix Ex Taq II, 0.8 μL upstream primers, 0.8 μL downstream primers, 7 μL DEPC water, 0.4 μL ROX II, and 1 μL cDNA. Reaction program: pre-degeneration at 95 °C for 3 min, degeneration at 95 °C for 10 s, annealing, and extension at 60 °C for 34 s, 40 cycles in total. The information of the relevant quantitative amplification primers is shown in Table 3. The primer sequences were synthesized by AuGCT Biology Co., Ltd.

**Table 3:** Relative quantitative PCR primers

Gene	Primers (5′ → 3′)
TNF-α	Forward: GGAATACCTGGACTATGCTGA
	Reverse: CCTCACTTCCCTACATCCCT
IFN-α	Forward: CAGCCTGGTCCTTACTCCTG
	Reverse: CTGCTCTGACAACCTCCCAG
IFN-β	Forward: CCAGATGGTTCTCCTGCTGTGT
	Reverse: GACCAATACGGCATCTTCCTTC
IL-1β	Forward: CCTTGGGTATCAGGGACAA
	Reverse: TGCGTATGGCTTTCTTTAGG
IL-10	Forward: GCTGTGCAGAAGTTCATGTT
	Reverse: GCTCAGGGAGGCCTCTTCAT
IL-6	Forward: AGAGGCACTGGCAGAAAAC
	Reverse: TGCAGGAACTGGATCAGGAC
GAPDH	Forward: GGTGATGCTGGTGCTGAGTA
	Reverse: TCATAAGTCCCTCCACGATG

### Data analysis

All in vitro experiments were repeated at least three times, and the results were consistent. The GraphPad Prism software was used for statistical analysis and mapping. Significance analysis was made through an independent sample *t *test, *P < 0.05 indicated significant differences between data and **P < 0. 01 indicated extremely significant differences between data.

## Results

### The concurrent immunization of GTP and PPR vaccines enhances the immune effect of the PPR vaccine

**Fig. 1:**
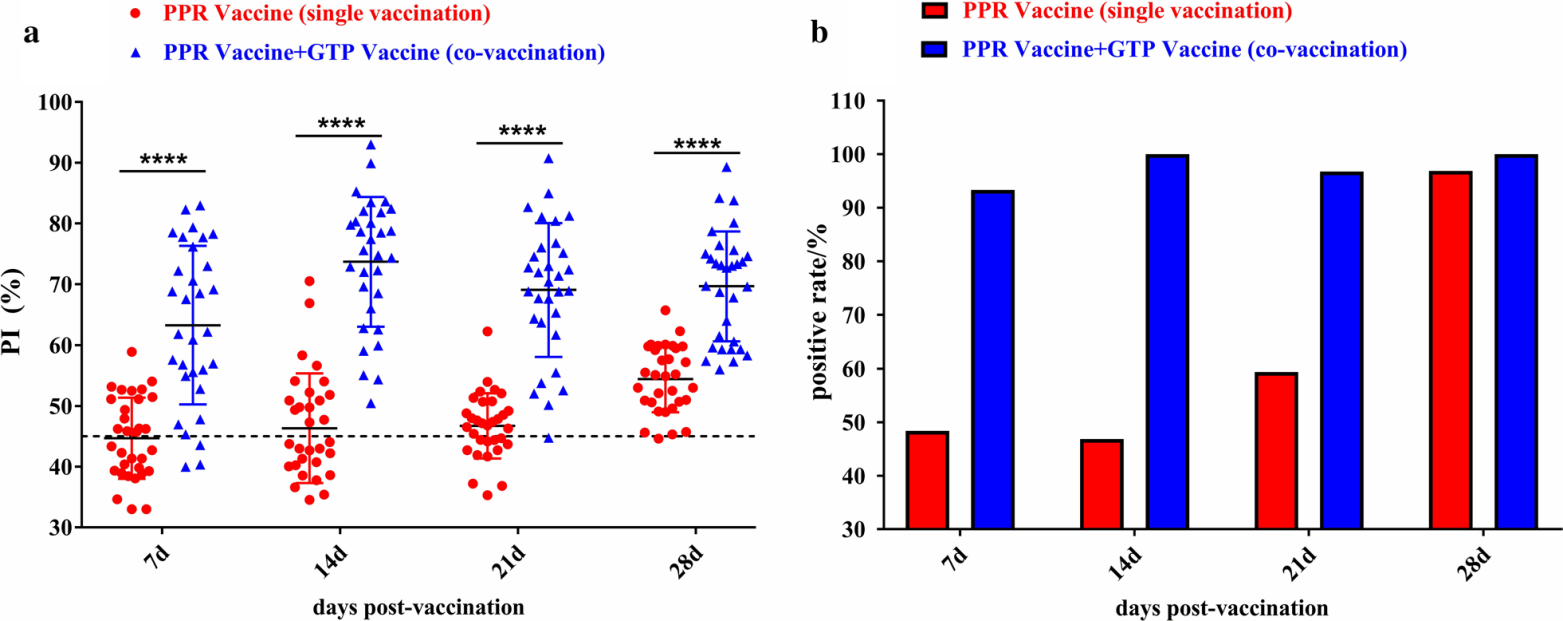
The concurrent immunization with GTP and PPR vaccines enhances the immune effect of the PPR vaccine. **a** The experimental sheep were vaccinated at 7d, 14d, 21d, and 28d to collect animal blood samples, centrifuge the blood at 4000 rpm for 8 min, and separate the serum samples. Detect PPR vaccine antibody PI values in group A (PPR vaccine single vaccination, red mark) and group C (PPR vaccine and GTP vaccine co-vaccination, blue mark) by Blocking ELISA Kit for Detecting Antibody of PPRV. Details of the groups are as in Table 1, "–" means Calculate Critical (cut off). **b** Calculate the positive rate of PPR vaccine by detecting the PI value of the antibody produced by the animals in groups A and C after being vaccinated with PPR vaccine. The red mark is the positive rate of PPR vaccine in the PPR vaccine single vaccination group, and the blue mark is the positive rate of PPR vaccine in the PPR vaccine and GTP vaccine co-vaccination group

After vaccination, the antibody levels in Group A (immunized with PPR vaccine alone) showed a gradual upward trend. On day 7, 14, and 21 after vaccination, 43.3%, 53.3%, 60% of sheep respectively, had a higher antibody level compared to the critical value (Fig. 1a). The antibody levels in Group C (immunized with GTP and PPR vaccines at the same time) were all higher than those in Group A, and the effect was the most significant on day 14 (70% of the sheep with higher antibody levels). Only day 7, four sheep had a lower antibody level compared to the critical value. The antibody levels of the sheep on day 21 and day 28 were consistent (Fig. 1a). The positive rate in Group A was on the rise and reached over 90% on day 28, while the positive rate in Group C was continuously stable and reached over 90% at all time points (Fig. 1b). As shown by the experimental data on the antibody levels and positive rate, the concurrent immunization of PPR and GTP vaccines induced higher antibody levels than the PPR vaccine alone, and the results for the positive rate were the same.

### The concurrent immunization of GTP and PPR vaccines inhibits the immune effect of the GTP vaccine

**Fig. 2:**
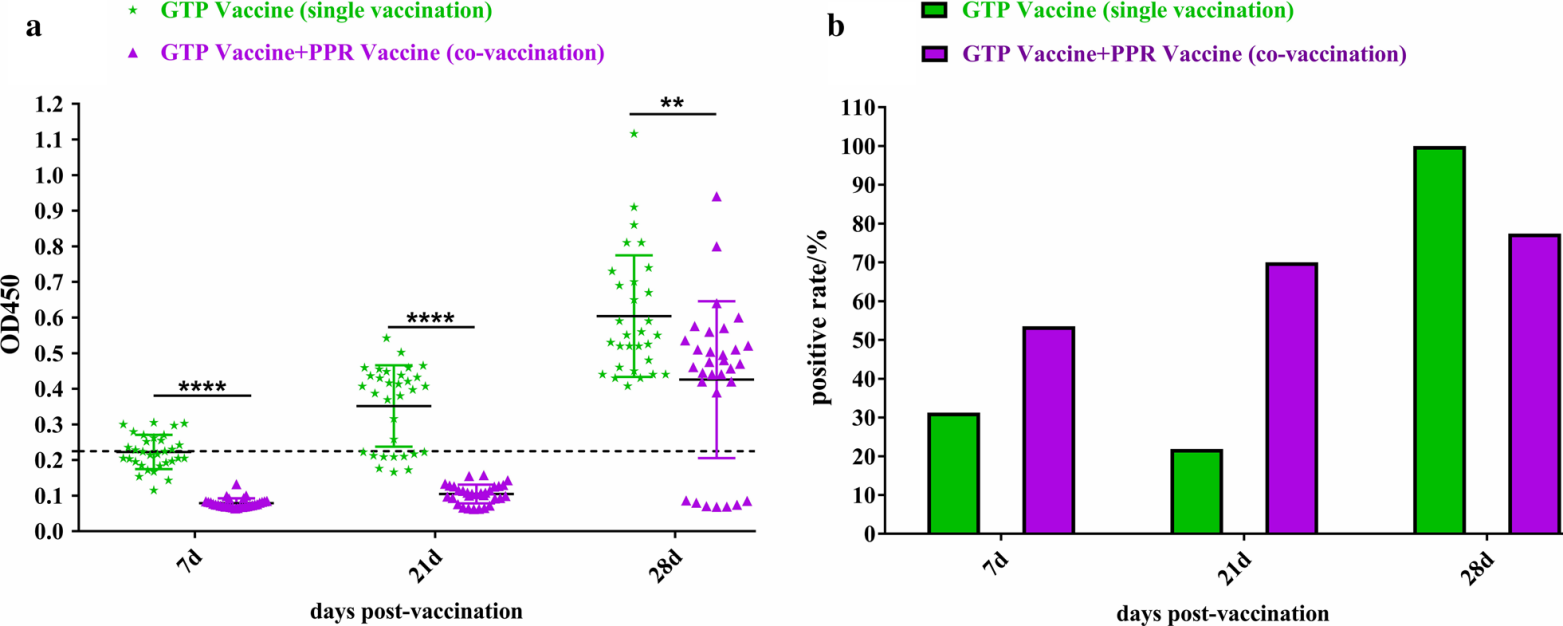
The concurrent immunization of GTP and PPR vaccines inhibits the immune effect of the GTP vaccine. **a** The experimental sheep were vaccinated on 7d, 21d, and 28d to collect animal blood samples, centrifuge the blood at 4000 rpm for 8 min, and separate the serum samples. The Inderict ELISA Kit for Detecting Antibodies of Capripox was used to detect the antibody level of GTP vaccine in group B (GTP vaccine single vaccination, green mark) and group C (GTP vaccine and PPR vaccine co-vaccination, purple mark). Details of the groups are as in Table 1, "–" means Calculate Critical (cut off). **b** Calculate the positive rate of GTP vaccine by detecting the results of the OD_450_ value of the antibody produced by the GTP vaccine after the animal vaccination in groups B and C. The green mark is the GTP vaccine positive rate in the GTP vaccine single vaccination group, and the purple mark is the GTP vaccine positive rate in the GTP vaccine and PPR vaccine co-vaccination group

After vaccination, the antibody levels in Group B (inoculated with GTP vaccine alone) showed a gradual upward trend. On day 7 after vaccination, the antibody OD values in only 8 sheep were greater than the critical value (0.25); on day 21, the antibody OD values in 70% of the sheep were greater than the critical value; on day 28, the antibody OD values of all sheep were greater than the critical value (Fig. 2a). The antibody levels in Group C (inoculated with GTP and PPR vaccines at the same time) were all lower than those in Group B. The antibody OD values of the sheep did not exceed the critical value on day 7 and day 21. On day 28, the antibody levels increased, and the antibody OD values of only 7 sheep did not reach the critical value (Fig. 2a). As shown by the positive rate results, on day 7 and day 21, the positive rate of the group immunized with both vaccines was higher by 70% compared to the group immunized with the GTP vaccine alone. On day 28, the positive rate in the group immunized with the GTP vaccine alone was higher than that in the group immunized with both vaccines, and the positive rate was 77% (Fig. 2b), which met the national standards. The experimental data on antibody levels and positive rate indicated that the antibody levels in sheep immunized with PPR and GTP vaccines were lower than in those immunized with GTP vaccine alone.

### Results of neutralizing antibody titer through VNT

**Fig. 3:**
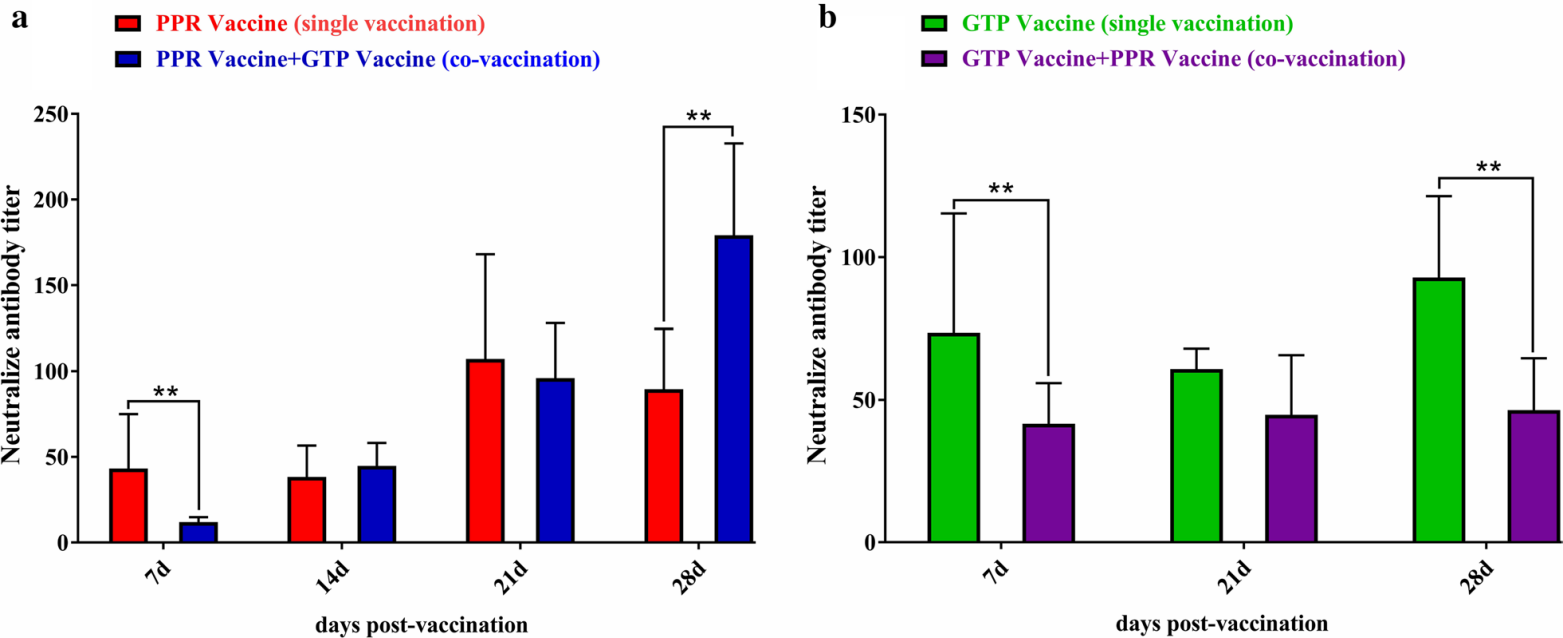
The neutralizing antibody titer of animals after vaccination was detected by the VNT method. **a** The virus neutralization test was used to detect the antibody titer of the PPR vaccine in group A (PPR vaccine single vaccination, red mark) and group C (PPR vaccine and GTP vaccine co-vaccination, blue mark). Details of the groups are as in Table 1. **b** The virus neutralization test was used to detect the antibody titer of the GTP vaccine in group B (GTP vaccine single vaccination, green mark) and group C (GTP vaccine and PPR vaccine co-vaccination, purple mark). Details of the groups are as in Table 1

After vaccination, the antibody titer in Group A (immunized with PPR vaccine alone) increased and reached the highest value on day 21, and the antibody titer decreased on day 28, but was still higher than that on day 7 and day 14. The antibody titer in Group C (immunized with PPR and GTP vaccines at the same time) was on the rise as a whole and reached the highest value on day 28. These experimental data showed that the antibody titer of the concurrent immunization with PPR and GTP vaccines was higher compared to the immunization with the PPR vaccine alone (Fig. 3a). After immunization, the antibody titer in Group B (immunized with GTP vaccine alone) underwent some changes and reached the highest value on day 28. After immunization, the antibody titer in Group C (immunized with PPR and GTP vaccines at the same time) was lower than that in Group B and remained stable at the same level. These experimental data suggested that the antibody titer of the concurrent immunization with PPR and GTP vaccines was lower than that of the immunization with the GTP vaccine alone (Fig. 3b).

### Virus titration of PPR and GTP vaccine strains through TCID_50_

**Fig. 4:**
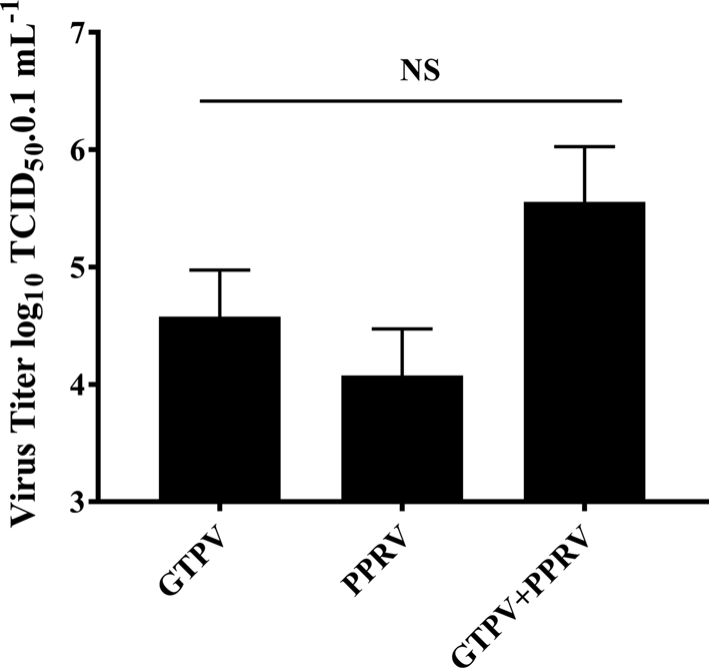
Virus titration of single infection with PPRV or GTPV alone or co-infection of PPRV and GTPV. Vero cells were infected with equal amounts of PPRV and GTPV, and 50% of the tissue-culture infective dose (TCID _50_) was used to detect the virus titer

**Fig. 5:**
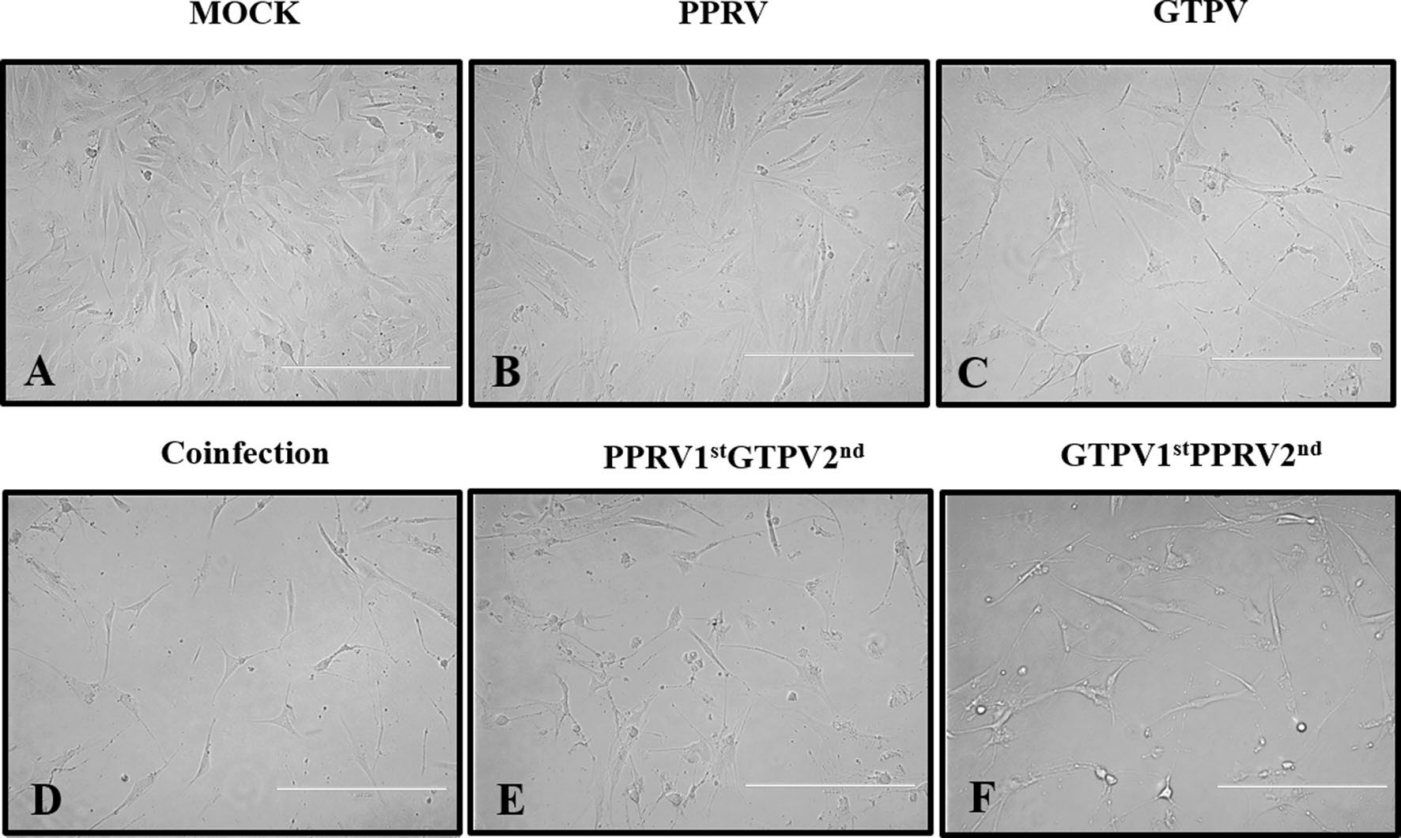
Images of monolayer Vero cells before and 72 h after the inoculation (× 400). Vero cells were infected with PPRV and GTPV at 0.5 MOI. An inverted fluorescence microscope was used to observe cellular pathological changes every 24 h, and photographic records were made after 72 h of observation. Details of the infection types are in as Table 2

**Fig. 6:**
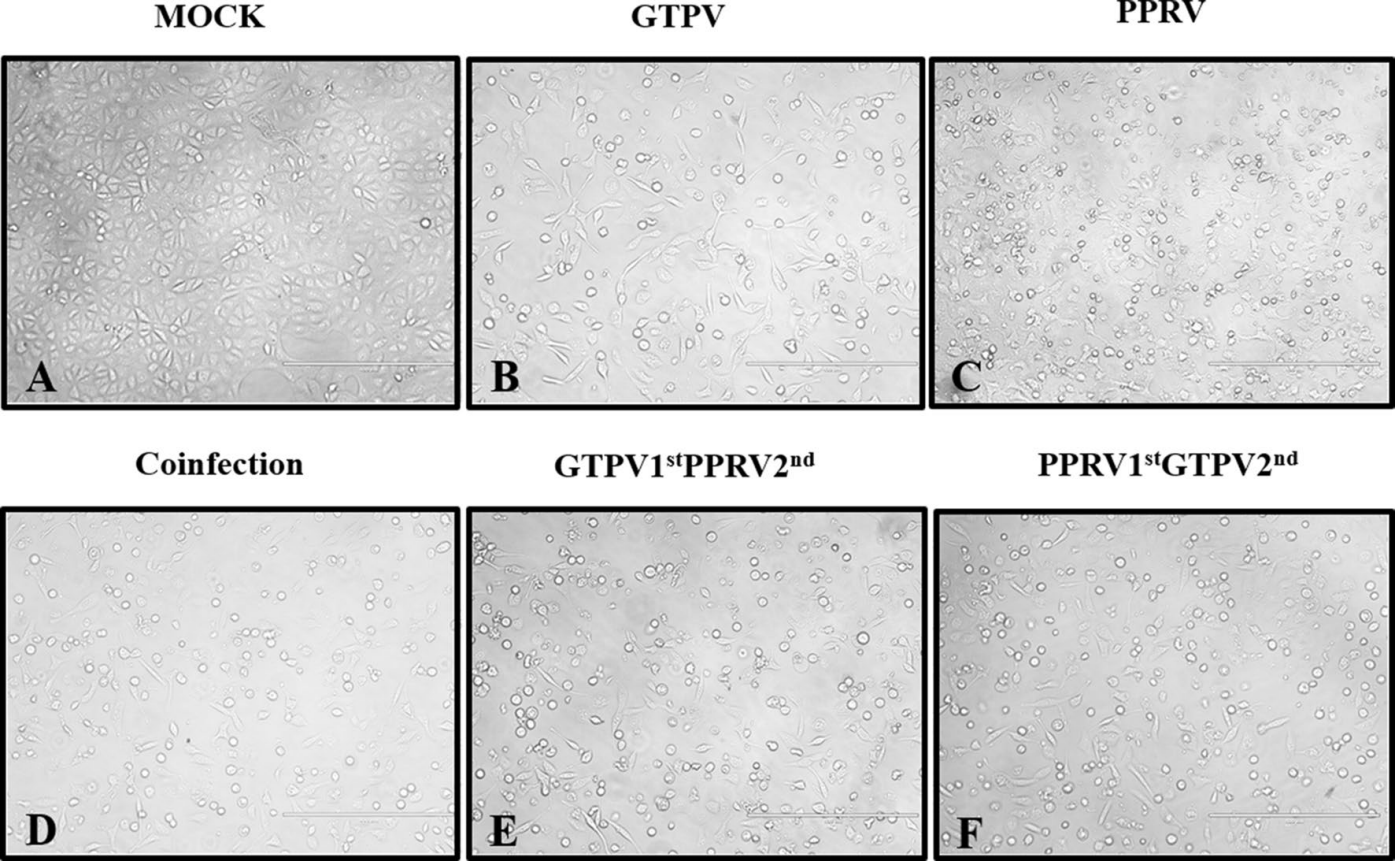
Images of monolayer GSF cells before and 72 h after the inoculation (× 400). GSF cells were infected with PPRV and GTPV at 0.5 MOI. An inverted fluorescence microscope was used to observe cellular pathological changes every 24 h, and photographic records were made after 72 h of observation. Details of the infection types are in as Table 2

TCID_50_ determination of PPRV and GTPV on Vero cells was performed by the Reed-Muench method (Fig. 4), and the results were as follows: TCID_50_ of attenuated GTP vaccine strains = 10^–4.62^/0.1 mL; TCID_50_ of attenuated PPR vaccine strains = 10^–4.092^/0.1 mL, TCID_50_ of the co-infection with both viruses = 10^–5.553^/0.1 mL. For each virus, Vero and GSF cells were infected with 0.5 MOI alone or in combination. Mixed viral infection indicated the inoculation of the two viruses (co-infection) or single inoculation at an interval of 3 h (repeated infection). The cells were observed every 24 h. At 72 h after the viral infection, the groups infected with PPRV or GTPV alone showed characteristic pathological changes, while the co-infection group had a cytopathic effect (CPE). In the co-infection group, CPE occurred earlier (60 h after infection), compared to the other two groups (72 h after infection) (Fig. 5), indicating that viral co-infection increases cytopathy. For GSF cells, the group infected with PPRV alone did not exhibit CPE, but the group infected with GTPV underwent typical CPE (Fig. 6).

### Viral nucleic acid load of GSF cells infected with PPR and GTP vaccine strains

**Fig. 7:**
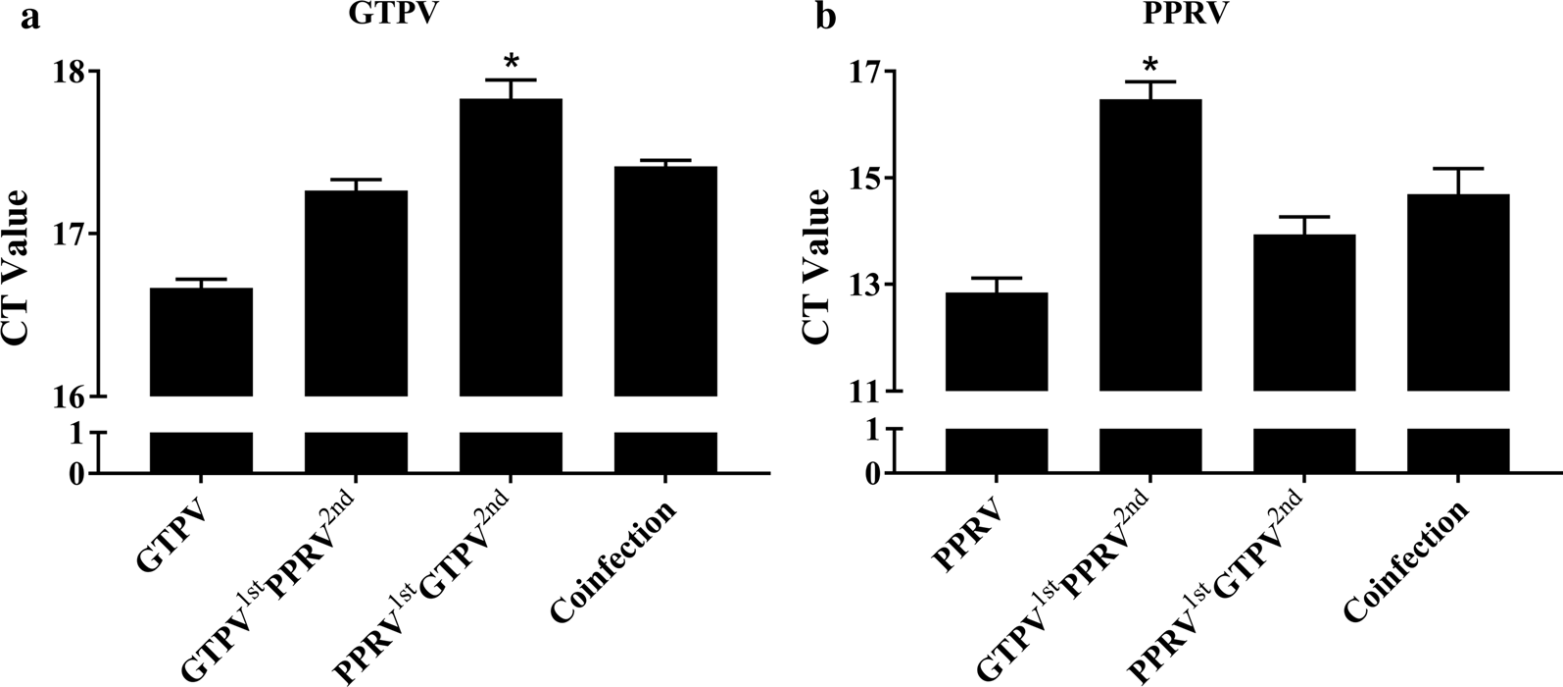
Quantification of viral nucleic acid in cells at 36 h after virus infection. **a** The viral nucleic acid load of GTPV after the GSF cells were infected. **b** The viral nucleic acid load of PPRV after the GSF cells were infected. Details of the infection types are in as Table 2. All data represent the mean SD, n = 3 for each group, two-tailed *t *test was used to calculate the significant difference, which is marked as *P < 0.05, **P < 0.01, ***P < 0.001

To explore the replication of PPRV and GTPV in host cells, real-time PCR was used to detect the changes in the viral load of the two viruses after infecting GSF cells. As shown in Fig. 7a, the viral load of GTPV in the group infected with GTPV alone was higher than that in the group co-infected with PPRV and GTPV. Among the groups co-infected with PPRV and GTPV, the group infected with PPRV followed by GTPV had the lowest rival load. As shown by the detection results in Fig. 7b, the viral load of PPRV in the group infected with PPRV alone was higher than in the remaining groups, and the group infected with GTPV followed by PPRV had the lowest viral load.

### Impact of GSF cells infected with viruses on the expression levels of cellular immune-related factors

**Fig. 8:**
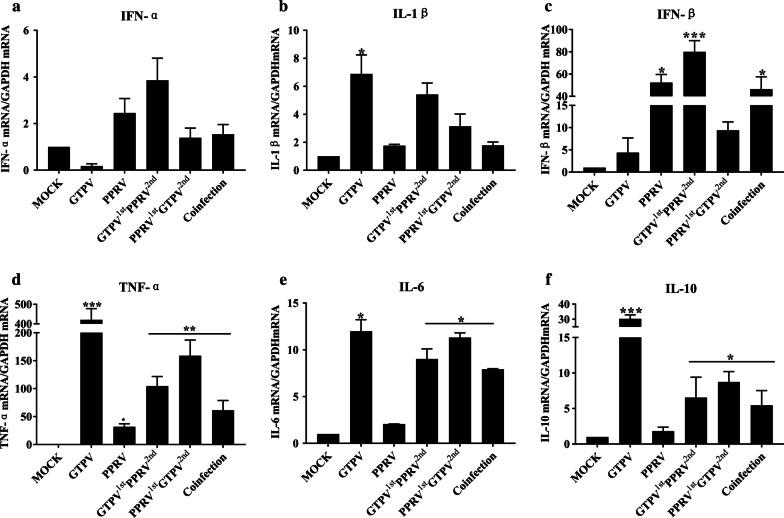
Detection of expression levels of cytokines after GSF cells were infected with viruses. GSF cells were infected with PPRV, GTPV, and a mixture of the two viruses (MOI = 0.5) for 36 h. The mRNA expression levels of IFN-α, IL-1β, IFN-β, TNF-α, IL-6, and IL-10 (**a**–**f**) were measured by RT-qPCR assay. All data represent the mean SD, n = 3 for each group, two-tailed *t *test was used to calculate the significant difference, which is marked as *P < 0.05, **P < 0.01, ***P < 0.001 in the figure

It remains unknown how the vaccine affects cytokines expression, and the role cytokines have in virus replication. We therefore detected and analyzed the cytokines through real-time PCR. The detection results are shown in Fig. 8a.

At 36 h after the viral infection, the IFN-α mRNA in the group infected with GTPV alone were lower than those in the control group. The group infected with GTPV followed by PPRV (PRV vaccination performed 3 h after GTP vaccination) had the highest IFN-α mRNA expression. Compared with the groups infected with a single virus, the IFN-α expressions in the co-infection groups was significantly enhanced. As shown in Fig. 8b, after the viral infection, the IL-1β mRNA expression in all groups was enhanced to varying degrees; the group infected with GTPV alone had the highest up-regulation, and the group infected with PPRV alone the lowest expression. Compared with the group infected with PPRV alone, the mRNA expression levels of IL-1β were increased in all the groups co-infected with PPRV and GTPV; and were decreased in the group infected with GTPV alone. Moreover, Fig. 8c showed that after the viral infection, IFN-β mRNA expression was enhanced in all infection groups to varying degrees; IFN-β mRNA in the group infected with GTPV followed by PPRV had the highest up-regulation (80 times higher than the control group), while the group infected with GTPV alone the lowest expression. Compared with the group infected with GTPV alone, the IFN-β mRNA expression in the co-infection groups was significantly enhanced. As shown in Fig. 8d–f, the mRNA expression levels of TNF-α, IL-6, and IL-10 were enhanced to varying degrees in all infection groups; the group infected with GTPV alone had the highest up-regulation, which was 420, 12, and 30 times higher compared to the control group, while the group infected with PPRV alone showed the lowest expression. Compared with the group infected with PPRV alone, the mRNA expression levels of TNF-α, IL-6 and IL-10 were enhanced in all co-infection groups. In particular, the group infected with PPRV followed by GTPV was the most significant, but compared with the group infected with GTPV alone, the mRNA expression levels of TNF-α, IL-6, and IL-10 in all co-infection groups were inhibited.

## Discussion

PPRV and GTPV are endemic diseases in the Middle East, Asia, and Africa and are highly contagious in small ruminants. The high morbidity and mortality accompanying these two diseases have caused devastating economic consequences [24, 25]. Mixed natural infections with PPRV and GTPV or PPRV and other poxviruses have also been reported in Asia and Africa [26, 27]. Dual or multiple infections caused by several pathogens further increased the morbidity and mortality of animals, within faunas, and between animals. In disease-endemic countries, vaccination is considered the only economically feasible method to control the disease and increase the productivity of small ruminants [28]. In order to reduce the cost of vaccination, there is a phenomenon that the two vaccines are used simultaneously in China. However, there are limited data on the specific interaction mechanism of PPRV-CaPV during the co-infection in animals.

In this study, we examined the mutual interference between the two vaccines in vivo and in vitro. For the animal experiments, sheep were immunized with a single or combined vaccines. The results showed that single immunization can achieve normal immune effect. This observation is consistent with most live attenuated vaccines. Simultaneous immunization of two vaccines enhances the antibody level of PPR, which is higher than most live attenuated vaccines produce higher levels of antibodies [29–31]. After vaccinating the animal with GTP and PPR vaccines, the antibody level of the GTP vaccine was reduced, and its immune effect was inhibited, but the antibody level increased over time (28 days after immunization); Moreover, no difference was found between the GTP + PPR group compared to the GTP vaccine alone group. This is inconsistent with the results of S. S. Chaudhary's research [29]. S. S. Chaudhary used the vaccinia RF strain to adapt to vero cells and prepared a combined vaccine with vero adapted PPRV, which was a mixture of the two vaccines and injected immunization. This may be the reason for the inconsistency with the results of this experiment. In this study, we also performed VNT to detect the antibody level, and we compared it with the ELISA. The results did somewhat differ compared to the ELISA results, but the general trend was the same. Of course, these differences might be due to the different methods used in the two experiments. A previous study used the glycoprotein on goat poxvirus-vectored PPRV as a recombinant vaccine, which could be used to vaccinate both important pathogens (PPRV and GTPV/SPPV) in small ruminants [32]. Yet, vaccines based on the recombinant GTPV vector might not produce a good antibody response due to preexisting vector immunity and might not be an ideal candidate vaccine. The results of this study clarified that PPR and GTP vaccines have mutual interference problems, but on the 28th day, the antibody level of the goat pox vaccine group recovered at the same time as the antibody level of the goat pox vaccine alone. Disadvantage of this study is that virus challenge experiment after immunization was not performed.

When two viruses infect the same host or tissue cells, there might be interference between the viruses and interaction between the viruses and cells. Viral interference is a phenomenon where one virus can inhibit the replication of the other [33–35]. In this study, we found that the co-infection with PPRV and GTPV aggravated the CPE of cells. Both viruses were able to proliferate in GSF cells, and the nucleic acid load in the group infected with PPRV or GTPV alone was the highest, For PPRV, co-infection inhibits the replication of the PPR vaccine strain, but enhances the immunity of the PPR vaccine, but it is different for GTPV. while other co-infection groups had conditional effects on the proliferation of PPRV and GTPV. This suggested that the two viruses competitively proliferate when inoculated on GSF cells, which was quite different from the results of other studies on viral interference [36]. Other studies found that under co-infection with PCV2 and Gram-negative bacteria, AIV, and NDV, the replication of only one virus was reduced [37, 38]. Furthermore, the replication rate of one virus was slightly higher, and that of the other virus was relatively lower [39, 40]. The differences in the obtained results could be explained by different experimental times, experimental settings (in vitro), different virus strains, and host cells used. There is currently no research on the impact of the co-infection with PPRV and GTPV on cell replication in vitro, making the comparison difficult to perform. This experiment showed that PPRV and GTPV inhibited each other. Nevertheless, whether the mutual interference between PPRV and GTPV maintains the same situation throughout the replication cycle or within a period of time needs to be further validated.

The balance between immune cells has a vital role in the immunity and pathogenesis of contagious diseases. The virus replication interferes with normal cellular processes and affects gene expression in cells. Therefore, cytokines' detection offers valuable reference information for the host's response to the infection and the nature of the inflammatory response. As the major proinflammatory cytokines, TNF-α, IL-1β, IL-6, and IFN-α are closely associated with the occurrence of inflammation and have a key role in coordinating and activating adaptive immune responses in diseases [41]. IL-10 is an immunosuppressive factor with multidirectional biological activity. Its overexpression can cause immunosuppression and reduce immunity. This study detected the expression levels of cytokines such as TNF-α, IL-1β, IL-6, IL-10, IFN-α, and IFN-β and found that after the viral infection, the expression levels of IL-1β, IL-6, IL-10, and IFN-α in the co-infection groups were significantly enhanced in contrast to the group infected with PPRV alone. Compared with the group infected with GTPV alone, the expressions of IL-1β, IL-6, IL-10, and IFN-α in the co-infection groups were inhibited. The up-regulated expression levels of IFN-β in the co-infection groups indicated that the co-infection with PPRV and GTPV promotes the viral survival rate by inhibiting the protective immune response. The experimental results in this study showed that co-infection could cause up-regulated expressions of many proinflammatory cytokines, thus inhibiting the other virus replication. This suggested a correlation between the expressions of inflammatory cytokines and virus replication. It can be speculated that viral infection can promote the mRNA expression of TNF-α, IL-1β, IL-6, IL-10, IFN-α, and IFN-β and might lead to the disorder of humoral immunity and cellular immunity, thereby regulating virus replication.

Collectively, this study confirmed the mutual interference between GPT and PPR vaccines in vivo; the concurrent use of both vaccines can enhance the PPR vaccine's immune effect and inhibit that of the GTP vaccine, hence indicating that the two vaccines cannot be used at the same time. We also found that GTP vaccine strains could inhibit the replication of PPR vaccine strains, while PPR vaccine strains enhanced GTP vaccine strains' replication in vitro. After the infection with GTP viruses (GTPV) and PPR viruses (PPRV), cytokines such as TNF-α, IL-1β, IL-6, IL-10, IFN-α, and IFN-β might regulate their replication in vitro.

## Conclusions

In summary, GTP vaccine and PPR vaccine strains interfered replication with each other visa cytokines such as TNF-α, IL-1β, IL-6, IL-10, IFN-α, and IFN-β, affecting the antibody levels when they used simultaneously. Compared with single vaccine vaccination. The next step is to evaluate the immune efficiency of these two vaccines when they used at the same time.

## Data Availability

All data generated or analyzed during this study are included in this submitted manuscript.

## Abbreviations

PPRV: Peste des petits ruminants virus
GTP: Goat pox virus
CPV: Capripoxvirus
IL: Interleukin
TNF-α: Tumor necrosis factor-α
IFN-γ: Interferon-γ
ELISA: Enzyme linked immunosorbent assay
VNT: Virus neutralization test
CPE: Cytopathic effect
MOI: Multiplicity of infection
TCID_50_: Median tissue culture infective dose
qPCR: Quantitative real-time polymerase chain reaction
DMEM: Dulbecco’s modified eagle’s medium
GSF: Goat skin fibroblast
FBS: Fetal bovine serum
OIE: The World Organisation for Animal Health
