# Correction to: In vitro and in vivo analyses of co‑infections with peste des petits ruminants and capripox vaccine strains

**DOI:** 10.1186/s12985-021-01576-2

**Published:** 2021-06-14

**Authors:** Dajun Zhang, Bo Yang, Ting Zhang, Xijuan Shi, Chaochao Shen, Haixue Zheng, Xiangtao Liu, Keshan Zhang

**Affiliations:** State Key Laboratory of Veterinary Etiological Biology, National Foot‑and‑Mouth Disease Reference Laboratory, Lanzhou Veterinary Research Institute, Chinese Academy of Agriculture Science, Lanzhou, 73004 People’s Republic of China

## Correction to: Virol J (2021) 18:69 10.1186/s12985-021-01539-7

Following publication of the original article [1], the authors have realized that there are a few errors as follows:

Figures 5 and 6, and respective legends, were erroneously swapped. The correct Figs. 5 and 6 are given below:

**Fig. 5:**
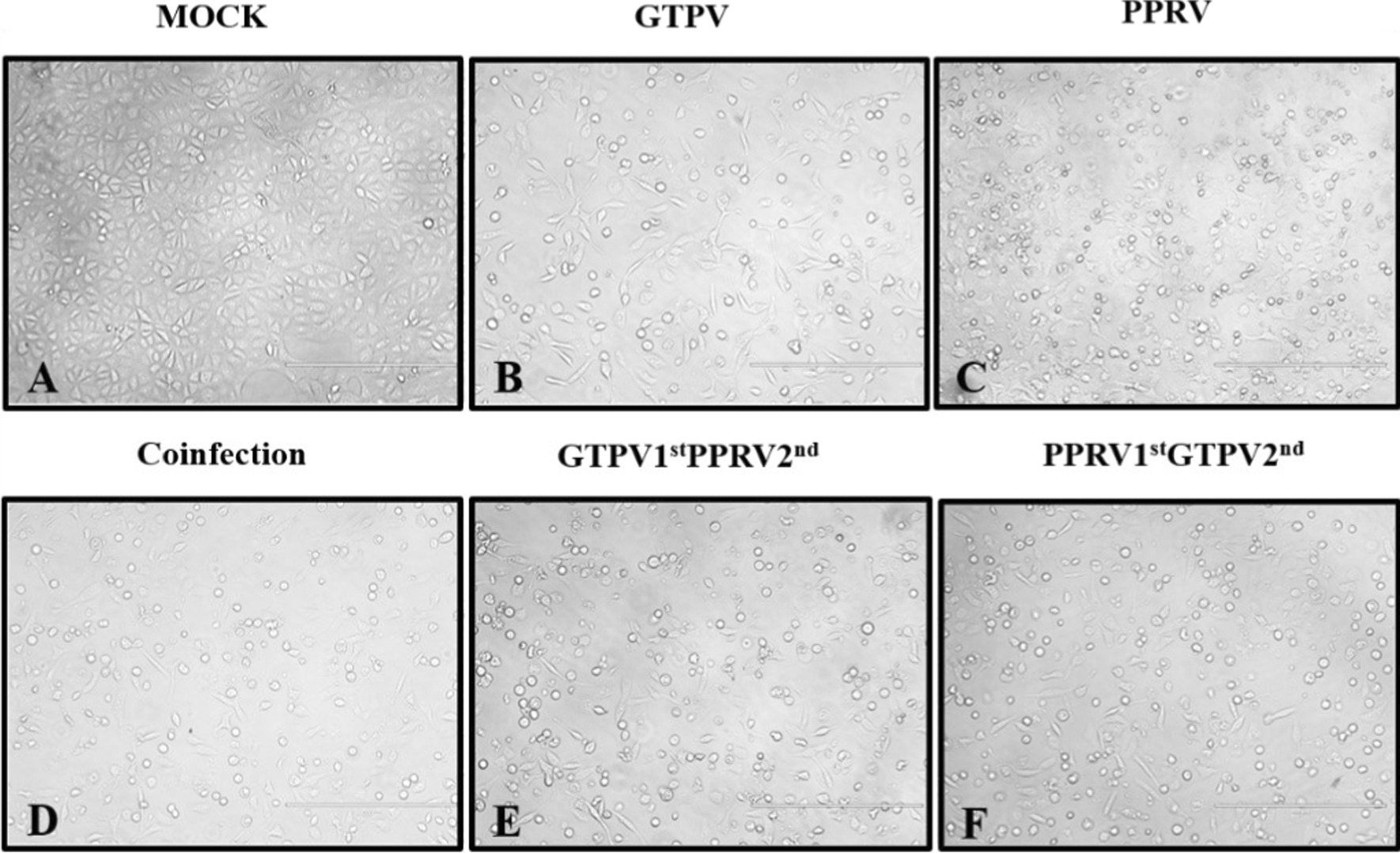
Images of monolayer Vero cells before and 72 h after the inoculation (400 ×). Vero cells were infected with PPRV and GTPV at 0.5 MOI. An inverted fluorescence microscope was used to observe cellular pathological changes every 24 h, and photographic records were made after 72 hof observation. Details of the infection types are in as Table 2

**Fig. 6:**
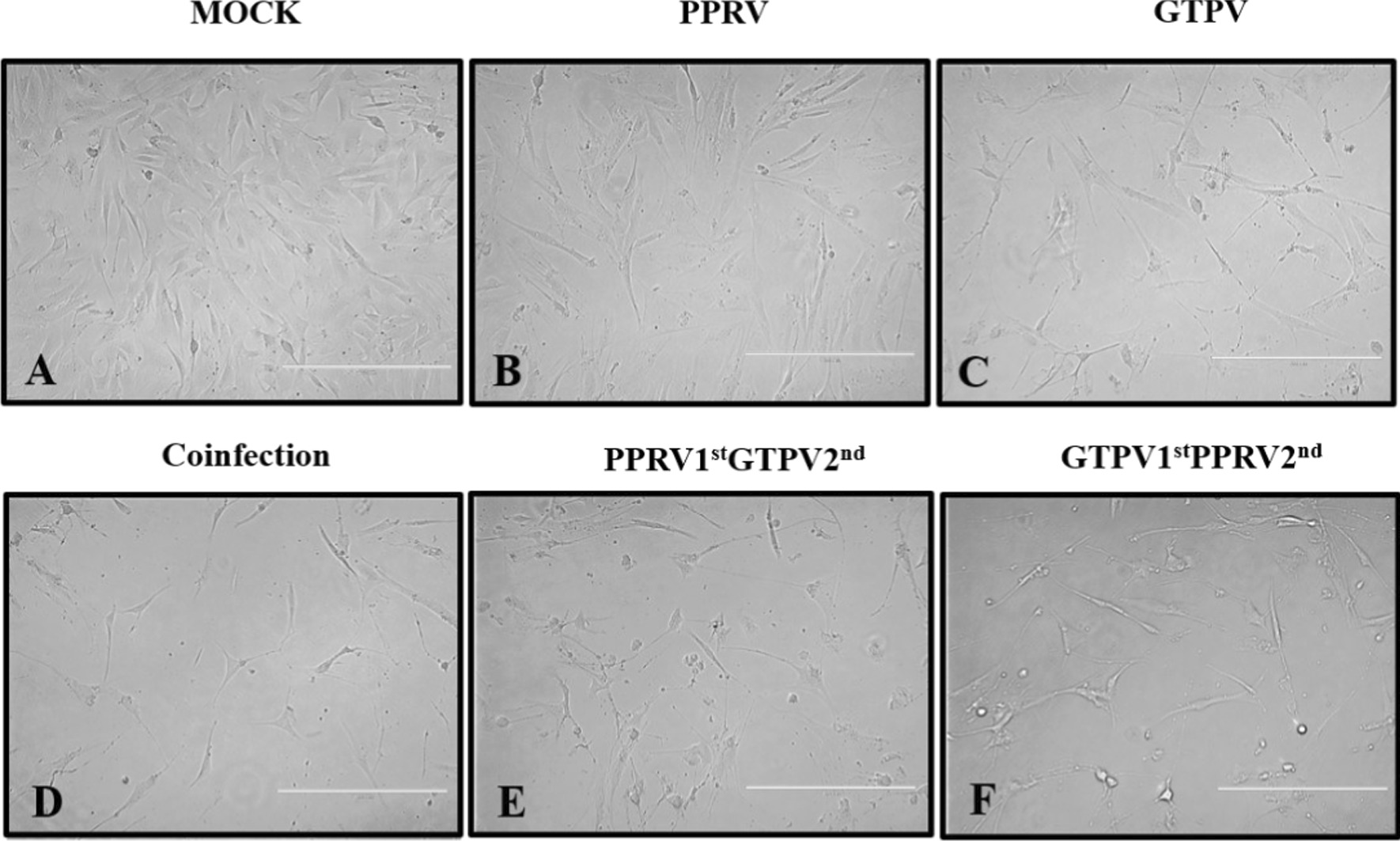
Images of monolayer GSF cells before and 72 h after the inoculation (400 ×). GSF cells were infected with PPRV and GTPV at 0.5 MOI. An inverted fluorescence microscope was used to observe cellular pathological changes every 24 h, and photographic records were made after 72 h of observation. Details of the infection types are in as Table 2

Table 2 has two clerical errors. The Correct Table 2 is given below:

**Table 2:** Cell monolayers were inoculated at a multiplicity of infection of 0.5 for each virus either singly (infection), or in combination

Infection type	Infection order		Infection MOI		Infection title
	First	Second	0 h	3 h	
Infection	PPRV		0.5	**–**	PPRV
Superinfection	PPRV	GTPV	0.5	0.5	PPRV 1st GTPV 2nd
Coinfection	PPRV + GTPV		1	**–**	Coinfection
Superinfection	GTPV	PPRV	0.5	0.5	GTPV 1st PPRV 2nd
Infection	GTPV		0.5	**–**	GTPV

Mixed viral infections resulted from inoculation with both viruses at the same time (coinfection) or from single inoculations occurring 3 h apart (superinfection)

“– “ means no virus infection

An error was identified in the Methods section. The updated Methods is given below and the changes have been highlighted in **bold typeface**.

## Methods

In this study, we vaccinated sheep with PPR and POX vaccines to compare the changes in the antibody levels between animals vaccinated with PPRV and POX vaccines alone and those co-infected with both vaccines simultaneously. The cell infection model was used to explore the interference mechanism between the vaccines in vitro. The antibody levels were detected with the commercial ELISA kit. **The Real-time Quantitative PCR method** was employed to detect the viral load changes and cytokines expression after the infection.

Two typing errors in the Discussion section. The updated Discussion is given below and the changes have been highlighted in **bold typeface**.

## Discussion

This study detected the expression levels of cytokines such as TNF-α, IL-1β, IL-6, IL-10, IFN-α, and IFN-β and found that after the viral infection, the expression levels of **IL-1β, IL-6, IL-10** in the co-infection groups were significantly enhanced in contrast to the group infected with PPRV alone. Compared with the group infected with GTPV alone, the expressions of IL-1β, IL-6, IL-10, and **TNF-α** in the co-infection groups were inhibited.

The authors confirm that this changes nothing in the conclusions of the article.

The original article [1] has been corrected.
